# Bevacizumab and irinotecan in recurrent malignant glioma, a single institution experience

**DOI:** 10.2478/raon-2014-0021

**Published:** 2015-03-03

**Authors:** Tanja Mesti, Maja Ebert Moltara, Marko Boc, Martina Rebersek, Janja Ocvirk

**Affiliations:** Department of Medical Oncology, Institute of Oncology Ljubljana, Ljubljana, Slovenia

**Keywords:** recurrent malignant glioma, bevacizumab, irinotecan, systemic therapy

## Abstract

**Background:**

Treatment options of recurrent malignant gliomas are very limited and with a poor survival benefit. The results from phase II trials suggest that the combination of bevacizumab and irinotecan is beneficial.

**Patients and methods.:**

The medical documentation of 19 adult patients with recurrent malignant gliomas was retrospectively reviewed. All patients received bevacizumab (10 mg/kg) and irinotecan (340 mg/m^2^ or 125 mg/m^2^) every two weeks. Patient clinical characteristics, drug toxicities, response rate, progression free survival (PFS) and overall survival (OS) were evaluated.

**Results:**

Between August 2008 and November 2011, 19 patients with recurrent malignant gliomas (median age 44.7, male 73.7%, WHO performance status 0–2) were treated with bevacizumab/irinotecan regimen. Thirteen patients had glioblastoma, 5 anaplastic astrocytoma and 1 anaplastic oligoastrocytoma. With exception of one patient, all patients had initially a standard therapy with primary resection followed by postoperative chemoradiotherapy. Radiological response was confirmed after 3 months in 9 patients (1 complete response, 8 partial responses), seven patients had stable disease and three patients have progressed. The median PFS was 6.8 months (95% confidence interval [CI]: 5.3–8.3) with six-month PFS rate 52.6%. The median OS was 7.7 months (95% CI: 6.6–8.7), while six-month and twelve-month survival rates were 68.4% and 31.6%, respectively. There were 16 cases of hematopoietic toxicity grade (G) 1–2. Non-hematopoietic toxicity was present in 14 cases, all G1-2, except for one patient with proteinuria G3. No grade 4 toxicities, no thromboembolic event and no intracranial hemorrhage were observed.

**Conclusions:**

In recurrent malignant gliomas combination of bevacizumab and irinotecan might be an active regimen with acceptable toxicity.

## Introduction

Malignant (high-grade) gliomas are rapidly progressive brain tumors comprising of anaplastic oligodendroglioma, anaplastic astrocytoma, mixed anaplastic oligoastrocytoma (all grade III, World Health Organization [WHO]) and glioblastoma (grade IV, WHO).^1^

The incidence of malignant gliomas is approximately 5/100,000. Malignant gliomas constitute 35–45% of primary brain tumors. Glioblastomas account for approximately 60 to 70% of malignant gliomas, while anaplastic astrocytomas represent 10 to 15%, and anaplastic oligodendrogliomas and anaplastic oligoastrocytomas 10% of malignant gliomas.^1^–^3^ The incidence of these tumors has increased slightly over past two decades, especially in the elderly. The peak incidence is in the fifth and sixth decade of life. The median age of patients at the time of diagnosis in the case of glioblastoma is 64 years and in the case of anaplastic gliomas is 45 years. Malignant gliomas are 40% more frequent in man than in woman and twice more frequent in white population than in black.^2^,^4^,^5^

In Slovenia from 1991 till 2005, a total of 1636 patients (878 males and 758 females) were diagnosed with brain cancer. Since 2001 till 2005 the microscopical verification was performed in 83% of cases: 82% were gliomas, of which two thirds were glioblastoma, 14% astrocytoma and 10% oligodendroglioma. Approximately 60% of the patients were diagnosed at age between 50 to 74 years, and 25% at age between 20 to 49 years.^6^

Glioblastoma tumors are gliomas of highest malignancy (grade IV), characterized by uncontrolled, aggressive cell proliferation and infiltrative growth within the brain and general resistance to conventional treatment. Despite the efforts to improve treatment outcome, the survival of patients with malignant gliomas is poor, with median survival of about 14 months.^7^,^8^ For glioblastoma, median time to progression after postoperative treatment with radiotherapy (RT) and temozolomide (TMZ) is 6,9 months and after RT alone 5.0 months.^9^ Although the 5-year OS analysis of the EORTC-NCIC trial has shown benefit for patients treated with RT and TMZ compared with only irradiated patients (9.8% vs. 1.9%), the median survival after progression remains only 6.2 months^10^, regardless of the initial treatment.

We present single institution experience of treating recurrent malignant gliomas with combination of bevacizumab and irinotecan.

## Patients and methods

We retrospectively reviewed medical documentation of 19 adult patients with recurrent malignant gliomas treated with bevacizumab and irinotecan at our center. All patients received bevacizumab at 10 mg/kg in combination with irinotecan 340 mg/m^2^ or 125 mg/m^2^ (with or without concomitant enzyme inducing antiepileptic drugs, respectively) every two weeks. Patient clinical characteristics (pre-recurrence treatment, performance status at the baseline), drug toxicities, response rate (RR), progression free survival (PFS) and overall survival (OS) were evaluated.

Statistical analysis was performed using the SPSS software, version 17.0. (SPSS Inc® Fulfillment center Haverhill MA, SPSS 17.0).

The study was approved by the Institutional Review Board Committe and was conducted in accordance with the Declaration of Helsinki.

## Results

### Patient characteristics

Fourteen (73.7%) males and five (26.3%) females were included. Median age was 44.7 years (range 27–74). Thirteen patients had glioblastoma, 5 anaplastic astrocytoma and 1 anaplastic oligoastrocytoma. In our group WHO performance status was 0–2. As an initial therapy all patients had a standard therapy with primary resection followed by postoperative chemoradiotherapy, with three-dimensional (3D) conformal radiation therapy (3D CRT), with 56 Gy in 28 fractions, combined with TMZ 75mg/m^2^, and followed with TMZ 150-200mg/m^2^ (4–20 cycles), except for one patient, with anaplastic astrocytoma of medullary cone, who had only biopsy.

### Treatment of recurrence

Bevacizumab and irinotecan systemic treatment was introduced after 1^st^, 2^nd^ and 3rd recurrence in 12 (63.2%), 5 (26.3%) and 2 (10.5%) patients, respectively (Table 1). No patient received additional RT at the time of disease recurrence.

### Efficacy

Average number of chemotherapy applications was 9.3 (range 1–17). Median follow up of the patients was 10 months (range 0–34 months). Radiological response was confirmed after 3 months in nine patients (1 complete response, 8 partial responses), seven patients had stable disease, and 3 patients have progressed. After 6 months, one patient remained in complete response, five had partial response, five had stable disease and eight have progressed (Table 2).

**FIGURE 1.**
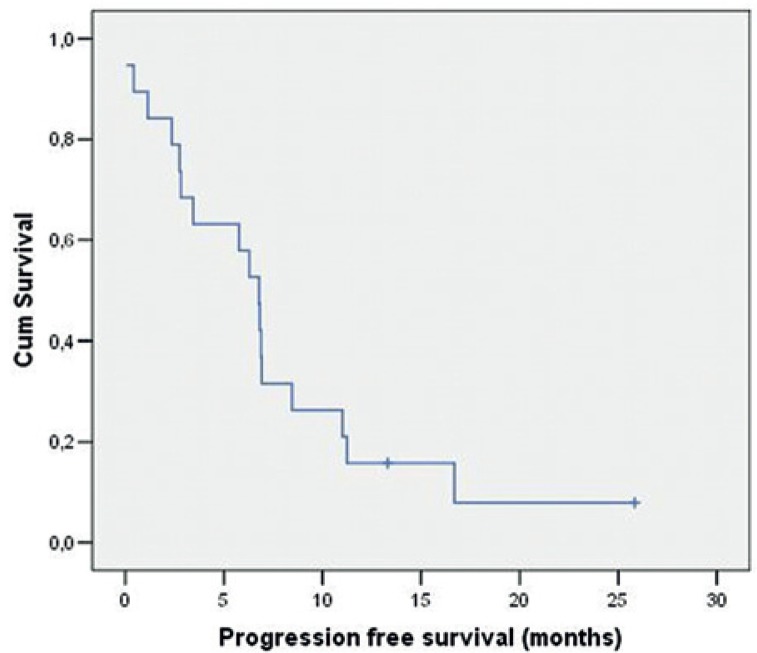
The median progression free survival (PFS).

**FIGURE 2.**
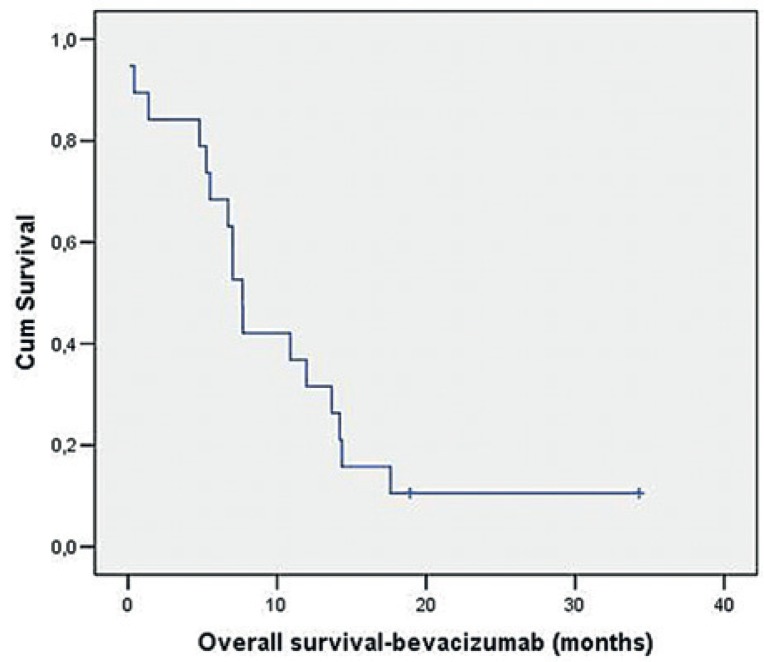
The median overall survival (OS).

The median PFS was 6.8 months (95% confidence interval [CI]: 5.3–8.3) (Figure 1) and the estimated six-month and twelve-month- PFS rates were 52.6% and 15.8%, respectively. The median OS was 7.7 months (95% CI: 6.6–8.7) (Figure 2), while the six-month and twelve-month survival rates were 68.4% and 31.6%, respectively.

### Toxicity

Toxicity was graded according to the National Cancer Institute Common Toxicity Criteria for Adverse Events (NCI-CTCAE) version 4.0. There were 16 cases of hematopoietic toxicity, only one patient had G2 neutropenia whereas in others only G1 toxic events were recorded. Two patients had neutropenia, six had lymphopenia, two had thrombocytopenia and one patient had anemia. No febrile neutropenia was observed. Nonhematopoietic toxicity G1-2 was present in 14 patients, with exception of one patient with proteinuria G3. Non-hematopoietic adverse events were: arterial hypertension (3), proteinuria (7), vomiting (2), diarrhea (1) and muscle pain (1). There was no G4 toxicity, no thromboembolic events and no intracranial hemorrhage observed (Table 3).

## Discussion

Everyday reality is that, despite efforts to improve therapies or to develop new ones, the outcome of treatment in malignant gliomas is poor with median survival of 14 months.^2^

The treatment of recurrent malignant gliomas with RT is controversial. Some data have suggested that fractionated stereotactic reirradiation (SRT) and stereotactic radiosurgery (SRS), which are also effective in another diseases, may be beneficial.^11^,^12^ Observational series of patients with recurrent malignant gliomas, treated with SRT showed the median survival of 16 months for patients with grade III tumors and eight months for those with grade IV lesions.^13^ The one-year survival rates were 65% and 23% for patients with grade III and IV lesions, respectively. Kong *et al*. in a patients with recurrent gliomas treated with SRS has achieved progression free survival for patients with grade III and grade IV gliomas of 8.6 and 4.6 months, respectively.^14^ All patients were treated with SRS treatments delivered by gamma knife, except for 5 patients treated by linear accelerator.

Chemotherapy treatment is more effective for anaplastic gliomas than for glioblastoma, and in general has modest value for recurrent malignant gliomas. There is no established chemotherapy regimen available and patients are best treated within investigational clinical protocols.

TMZ was evaluated in a phase II study in patients with recurrent anaplastic gliomas who had previously been treated with nitrosoureas.^15^ The response rate was 35%, and the 6-month PFS rate was 46%, comparing favorably with the 6-month PFS rate of 31% for therapies that were generally considered ineffective.^16^ In patients with recurrent glioblastoma, TMZ has only limited activity, with response rate of 5.4% and 6-month rate of PFS of 21%.^17^

Other chemotherapeutic agents that are used for recurrent gliomas include nitrosoureas, carboplatin, procarbazine, irinotecan, and etoposide. Nitrosoureas (carmustine, fotemustine) either as single agents or in combination regimens as procarbazine, lomustine and vincristine (PCV) have shown activity in phase II studies in previously treated patients. Brandes AA *et al*., conducted a phase II study on 40 patients with recurrent glioblastoma following surgery and standard radio-therapy, treated with carmustine as monotherapy. Median time to progression was 13.3 weeks and progression-free survival at 6-months was 17.5%.^18^ Schmidt F *et al*., has applied PCV, as combined regimen, to 86 patients with recurrent glioblastoma. There were three partial responses, but no complete responses. Median progression-free survival was 17.1 weeks and progression-free survival at 6 months was 38.4%.^19^

Bevacizumab is a monoclonal antibody, which binds to VEGF, the key driver of neovascularization, and thereby inhibits the binding of VEGF to its receptors, VEGFR-1 and VEGFR-2, on the surface of endothelial cells.

Anti-VEGF therapy, including bevacizumab, acts by binding to VEGF and preventing its cellular effects. However, this linear interaction represents only a partial view of the pathobiology of the disease and treatment processes. Consequently, the classical concept of linear interactions is being replaced by the concept of networks of interactions, emphasizing the importance of interactions between different components of a biologic system.^20^

In phase II studies Bevacizumab as a single agent or in combination with chemotherapy agents such as irinotecan demonstrated clinical activity for patients with grade 3 and grade 4 malignant gliomas (higher objective response, progression-free survival and overall survival) in recurrent glioblastoma^21^–^32^ and in May 2009, it has been approved by FDA for the secondary treatment of glioblastoma in USA^32^, but it is not approved yet by EMA in Europe.^34^

According to the meta-analysis of phase II trials with bevacizumab and irinotecan treatment in recurrent malignant gliomas^35^, which included 411 patients, the median progression-free survival time ranged from 2.4 to 13.4 months and the median overall survival time ranged from 6.2 to 14.9 months, with response rates ranging from 28% to 86%. The improvement in tumor response rate observed in patients with reccurent malignant gliomas treated with bevacizumab and irinotecan combination to those on other systemic drugs protocols was highly statistically significant (P = 0.00002), and so was the same with the OS (P = 0.024).

The most extensive experience with bevacizumab comes from a noncomparative phase II trial, in which 167 patients with recurrent glioblastoma, previously treated with chemotherapy with temozolomide were randomly assigned to bevacizumab, either as a single agent or at the same dose in conjunction with irinotecan.^24^ Treatment cycles were repeated every two weeks. The objective response rates with bevacizumab alone or in combination with irinotecan were 28% and 38%, respectively, the 6-month PFS rates were 43% and 50%, respectively and mOS times were 9.2 and 8.7 months, respectively. An update of the results was presented at the 2010 American Society of Clinical Oncology meeting.^23^ Overall safety and efficacy were similar to that previously presented; the 12 and 24-month survival rates were 38% and 16% to 17% on both treatment arms, which appear to be better than historical control series.

We treated 19 patients with recurrent malignant gliomas with bevacizumab and irinotecan, from August 2008 to November 2011. The objective response rates were 47.4% and 31.6% after 3 and 6 months respectively. The 6-month PFS and OS rate and interval were 52.6% and 68.4% and 6.8 and 7.7 months, respectively. One third of the patients (31.6%) reached twelve-month OS. Regarding toxicity, 78.9% patients experienced hematopoietic toxicity G1, with only one patient experiencing G2 neutropenia. As for the non-hematopoietic toxicity, 42.1% patients had adverse events G1, 26.3% G2 and one patient had G3 proteinuria. There were no grade 4 toxicities, no febrile neutropenia, no thromboembolic event and no intracranial hemorrhage observed. Comparison of our data with other studies is presented in the Table 4.

## Conclusions

In patients with recurrent malignant gliomas the combination of bevacizumab and irinotecan shows promising activity with acceptable toxicity although survival outcome is far from desired. Our results of the treatment of patients with recurrent malignant gliomas, regarding response rate, PFS and OS are comparable with the previously published data.

As all data about efficacy and safety of bevacizumab and irinotecan therapy in recurrent malignant gliomas are coming from phase II trials, larger phase III randomized controlled studies comparing bevacizumab plus irinotecan with other treatment protocols are warranted so that the efficacy can be assessed properly.

## Figures and Tables

**TABLE 1.** Systemic treatment of choice after disease progression

**Recurrence**	**Cht (number of patients)**
**First**	**Bevacizumab + Irinotecan 12 (63.2%)**
	BCNU 2
	TMZ 4
	PCV 1
**Second**	**Bevacizumab + Irinotecan 5 (26.3%)**
	BCNU 1
	PCV 1
**Third**	**Bevacizumab + Irinotecan 2 (10.5%)**

BCNU = Carmustine (BCNU), 80 mg/m2 BCNU on days 1 on 3; Cht = chemotherapy; PCV = lomustine (CCNU), 110 mg/m2, on day 1; procarbazine, 60 mg/m2 on days 8 to 21; and vincristine, 1.5 mg/m2 (maximum dose, 2 mg), on days 8 and 29; TMZ = temozolomide, 150–200 mg/m2/day on days 1 to 5 of each subsequent 28-day cycle

**TABLE 2.** Radiological response after 3 months and 6 months of concomitant irinotecan and bevacizumab treatment

**Response (n=19)**	**after 3 months**	**after 6 months**
**Complete Response (CR)**	1	1
**Partial Response (PR)**	8	5
**Stable Disease (SD)**	7	5
**Progression of Disease (PD)**	3	8

**TABLE 3.** Adverse events

	**Without AE n (%)**	**G1** **n (%)**	**G2** **n (%)**	**G3** **n (%)**	**G4** **n (%)**
**Hematopoietic toxicity**					
**leukopenia**	16 (84)	2 (10.5)	1 (5.3)	0	0
**neutropenia**	17 (89.5)	1 (5.3)	1 (5.3)	0	0
**lymphopenia**	13 (68.4)	6 (31.6)	0	0	0
**thrombocytopenia**	17 (89.5)	2 (10.5)	0	0	0
**anemia**	16 (84.2)	3 (15.8)	0	0	0
**febrile neutropenia**	0	/	/	/	0
**Non-hematopoietic toxicity**					
**arterial hypertension**	16 (84.2)	2 (10.5)	1 (5.3)	0	0
**proteinuria**	12 (63.2)	3 (15.8)	3 (15.8)	1 (5.2)	/
**sepsis**	0	/	/	/	0
**diarrhea**	18 (94.7)	1 (5.3)	0	0	0
**vomiting**	17 (89.5)	1 (5.3)	1 (5.3)	0	0
**muscle pain**	18 (84.7)	1 (5.3)	0	/	/
**intracranial hemorrhage**	0	0	0	0	0
**tromboembolic event**	0	0	0	0	0

0 = no adverse side effects; / = adverse events of this grade doesn’t exist

**TABLE 4.** Comparison of our study data with other studies

	**Patients**	**Response rate**	**PFS at 6 months**	**Median survival (months)**
**Bev+Irinotecan(Vredenburgh)**^25^	35	57%	46%	9.7
**Bev→Bev+Irinotecan (Kreisl)**^21^	85	35%	29%	7.2
**Bev+Irinotecan (Friedman)**^22^	167	28% / 38%	43% / 53%	9.2 / 8.7
**Bev+Irinotecan (IO Lj study)**	19	47.4%	52.6%	7.7

IO Lj = Institute of Oncology Ljubljana
